# Responder analysis of a randomized comparison of the 13.3 mg/24 h and 9.5 mg/24 h rivastigmine patch

**DOI:** 10.1186/s13195-014-0088-8

**Published:** 2015-03-08

**Authors:** José L Molinuevo, Lutz Frölich, George T Grossberg, James E Galvin, Jeffrey L Cummings, Tillmann Krahnke, Christine Strohmaier

**Affiliations:** Alzheimer’s Disease and Other Cognitive Disorders Unit, ICN, Hospital Clínic i Universitari, IDIBAPS, Villarroel 170, Barcelona, 08036 Spain; Department of Geriatric Psychiatry, Central Institute of Mental Health, Medical Faculty Mannheim, University of Heidelberg, Mannheim, Germany; Department of Neurology & Psychiatry, School of Medicine, St Louis University, St Louis, MO USA; Center for Cognitive Neurology, New York University Langone School of Medicine, New York, NY USA; Cleveland Clinic Lou Ruvo Center for Brain Health, Las Vegas, NV USA; Cleveland Clinic Lou Ruvo Center for Brain Health, Cleveland, OH USA; Novartis Pharma AG, Basel, Switzerland

## Abstract

**Introduction:**

OPtimizing Transdermal Exelon In Mild-to-moderate Alzheimer’s disease (OPTIMA) was a randomized, double-blind comparison of 13.3 mg/24 h versus 9.5 mg/24 h rivastigmine patch in patients with mild-to-moderate Alzheimer’s disease who declined despite open-label treatment with 9.5 mg/24 h patch. Over 48 weeks of double-blind treatment, high-dose patch produced greater functional and cognitive benefits compared with 9.5 mg/24 h patch.

**Methods:**

Using OPTIMA data, a *post-hoc* responder analysis was performed to firstly, compare the proportion of patients demonstrating improvement or absence of decline with 13.3 mg/24 h versus 9.5 mg/24 h patch; and secondly, identify predictors of improvement or absence of decline. ‘Improvers’ were patients who improved on the Alzheimer’s Disease Assessment Scale–cognitive subscale (ADAS-cog) by ≥4 points from baseline, and did not decline on the instrumental domain of the Alzheimer’s Disease Cooperative Study–Activities of Daily Living scale (ADCS-IADL). ‘Non-decliners’ were patients who did not decline on either scale.

**Results:**

Overall, 265 patients randomized to 13.3 mg/24 h and 271 to 9.5 mg/24 h patch met the criteria for inclusion in the intention-to-treat population and were included in the analyses. Significantly more patients were ‘improvers’ with 13.3 mg/24 h compared with 9.5 mg/24 h patch at Weeks 24 (44 (16.6%) versus 19 (7.0%); *P* < 0.001) and 48 (21 (7.9%) versus 10 (3.7%); *P* = 0.023). A significantly greater proportion of patients were ‘non-decliners’ with 13.3 mg/24 h compared with 9.5 mg/24 h patch at Week 24 (71 (26.8%) versus 44 (16.2%); *P* = 0.002). At Week 48, there was a trend in favor of 13.3 mg/24 h patch. Functional and cognitive assessment scores at double-blind baseline did not consistently predict effects at Weeks 24 or 48.

**Conclusion:**

More patients with mild-to-moderate Alzheimer’s disease who are titrated to 13.3 mg/24 h rivastigmine patch at time of decline are ‘improvers’ or ‘non-decliners’ i.e. show responses on cognition and activities of daily living compared with patients remaining on 9.5 mg/24 h patch.

**Trial registration:**

Clinicaltrials.gov identifier: NCT00506415; registered July 20, 2007.

**Electronic supplementary material:**

The online version of this article (doi:10.1186/s13195-014-0088-8) contains supplementary material, which is available to authorized users.

## Introduction

The cholinesterase inhibitors (ChEIs), galantamine, donepezil and rivastigmine, remain the predominant treatment for providing symptomatic relief for patients with mild-to-moderate Alzheimer’s disease (AD). To ensure the best outcome for the patient, it is important that use of the available therapies is optimized over the course of disease progression; suboptimal dosing of ChEI therapy may be associated with a reduced chance of response [1]. Patients who ‘respond’ to treatment are generally regarded as those who demonstrate a clinically meaningful benefit on an outcome measure. In neurodegenerative diseases, a treatment response refers to short-term improvement, longer-term stabilization or a slowed decline in one or more clinically relevant symptom domains [2].

The degree of impairment associated with AD is measured by evaluation of cognitive factors, such as learning and memory. The Alzheimer’s Disease Assessment Scale–cognitive subscale (ADAS-cog) [3] is the standard neuropsychological measure in trials involving patients with mild-to-moderate AD [4,5]. It is important that functional decline is also measured to assess the ability to perform clinically meaningful activities of daily living (ADL). The Alzheimer’s Disease Cooperative Study (ADCS)-ADL scale is among the instruments most commonly used to assess function in AD-type dementia [6,7].

In clinical trials, outcomes may be measured as: the mean difference in the total score on a given scale between active treatment and comparator groups; the delay to reaching specific milestones; or the percentage of ‘responders’ to treatment compared with a comparator treatment group or placebo. The criterion usually applied for a ‘responder’ is a four-point or more improvement on the ADAS-cog [8], as this is generally accepted to represent a clinically relevant change on an individual basis in patients with mild-to-moderate AD [9]. In addition, for a population of patients who, without treatment, display continuous decline, achieving any improvement or temporary stability on the ADAS-cog and other assessments may represent a significant therapeutic benefit. Therefore, it is important to assess not just those who improve on the ADAS-cog, but also those who do not decline. On the ADCS-ADL, a responder can be defined as a patient who improves or is stabilized in the course of a randomized clinical trial. It is also important to identify factors that may influence the likelihood of receiving a benefit from treatment, as patients may vary in their response to treatment according to their clinical characteristics [10,11].

Here, we present a *post-hoc* responder analysis of the instrumental domain of the ADCS-ADL scale (ADCS-IADL) data and the ADAS-cog data from the OPtimizing Transdermal Exelon In Mild-to-moderate Alzheimer’s disease (OPTIMA) clinical study (clinicaltrials.gov identifier NCT00506415). The aim of this analysis was firstly to identify the proportion of patients who demonstrated improvement or absence of decline with the 13.3 mg/24 h (15 cm^2^) versus the 9.5 mg/24 h (10 cm^2^) rivastigmine patch and, secondly to identify which patient characteristics are predictive of treatment response or non-decline.

## Methods

### Study population and study design

Detailed methodology of the OPTIMA study has been published previously [12]. Briefly, eligible patients were 50- to 85-years old, with a diagnosis of mild-to-moderate, probable AD according to the criteria of the National Institute of Neurological and Communicative Disorders and Stroke, and the Alzheimer’s Disease and Related Disorders Association (NINCDS/ADRDA) [13] and a Mini-Mental State Examination (MMSE) score of ≥10 and ≤24 [14].

Patients who met pre-specified functional decline criteria (per physicians’ judgment) and cognitive decline criteria (≥3 point decline from baseline, or ≥2 point decline from the previous visit, in MMSE score) during up to 48 weeks of initial open-label (IOL) treatment with the 9.5 mg/24 h rivastigmine patch, subsequently entered a 48-week double-blind (DB) phase of the trial. In the DB phase, patients were randomized to treatment with either the 13.3 mg/24 h or the 9.5 mg/24 h rivastigmine patch [12]. The OPTIMA study protocol was reviewed by the representative ethics committee for each participating center (see Additional file 1). The study was designed and implemented in accordance with Good Clinical Practice and the local regulations and ethical principles laid down in the Declaration of Helsinki [12]. All patients, or a legally acceptable representative, and caregivers provided written informed consent prior to participating in the study.

Of 1,584 patients enrolled into the IOL phase of the study, 567 met the pre-specified decline criteria during the IOL phase and were randomized into the DB phase; 280 to the 13.3 mg/24 h patch and 287 to the 9.5 mg/24 h patch [12]. The mean MMSE score ± standard deviation (SD) at baseline of the IOL phase, for those patients who declined during the IOL phase and, hence, were randomized into the DB phase, was 16.9 ± 3.6. At DB-baseline (DB-BL), the mean ± SD MMSE score was 14.1 ± 4.8 in the 13.3 mg/24 h patch group and 14.2 ± 4.6 in the 9.5 mg/24 h patch group, indicating that this represented a declining population [12].

### Study outcomes and responder definition

The co-primary outcomes of the OPTIMA study were the change from DB-BL to Week 48 in ADAS-cog and ADCS-IADL scores. In this *post-hoc* analysis, responder analyses were conducted in each treatment group, on data collected during the DB-treatment phase, by applying definitions for ‘improvement’ and ‘non-decline’ using ADAS-cog and ADCS-IADL criteria, in order to assess outcomes of both cognition and functional response. The percentages of patients with change from DB-BL in each responder category at Weeks 24 and 48 were compared between treatment groups (13.3 mg/24 h versus 9.5 mg/24 h patch).

On the ADAS-cog, where an increase in points represents decline: a clinically relevant improvement is assessed as a reduction in ADAS-cog score from DB-BL of four points or more; a lack of decline includes patients with clinically relevant improvements from DB-BL, non-clinically relevant improvements from DB-BL, or no change from DB-BL (that is, a reduction or no change from DB-BL in ADAS-cog score).

On the ADCS-IADL, where a decrease in points represents decline: a lack of decline is assessed as no change or any improvement from DB-BL (that is, change from DB-BL ≥0 points).

For the combined analysis, two groups were identified: those who improved on ADAS-cog and did not decline on ADCS-IADL (‘improvers’) and a wider group who did not decline on either scale (‘non-decliners’, which encompasses the ‘improvers’). Therefore, an ‘improver’ was defined as a patient who experienced an improvement from DB-BL in ADAS-cog score ≥4 points *and* no decline or a change from DB-BL in ADCS-IADL score ≥0 points; a ‘non-decliner’ was defined as a patient who experienced no decline or a change from DB-BL in ADAS-cog score ≤0 points *and* no decline or a change from DB-BL in ADCS-IADL score ≥0 points. Predictors of these combined response outcomes (‘improvers’ and ‘non-decliners’) were investigated at Weeks 24 and 48.

### Analysis

Patient data were analyzed according to the randomized treatment (13.3 mg/24 h or 9.5 mg/24 h patch). The primary analysis of this study was based on the intention-to-treat population in the DB phase (ITT-DB) using a last-observation-carried-forward (LOCF) imputation. The ITT-DB population with an LOCF imputation consisted of all patients who received at least one dose of study drug who also had at least one post-randomization assessment for both the ADAS-cog and ADCS-IADL.

Predictors of a combined response were investigated using a stepwise logistic regression model on both the combined criteria for improvement (‘improvers’) and the combined criteria for response (‘non-decliners’), with the following potential explanatory variables in addition to treatment: gender, ADAS-cog score at DB-BL, ADCS-IADL score at DB-BL, MMSE score at DB-BL of ≤12, and body weight category (<50 kg, 50 to 80 kg, >80 kg). Variables included in the final model were those with a *P*-value <0.15. Odds ratios (ORs) were calculated.

## Results

### Study population

In total, 265 patients randomized to the 13.3 mg/24 h patch and 271 patients randomized to the 9.5 mg/24 h patch met the criteria for inclusion in the ITT-DB population and were included in the present analysis.

### Response on the ADAS-cog and ADCS-IADL when analyzed separately

**Figure 1:**
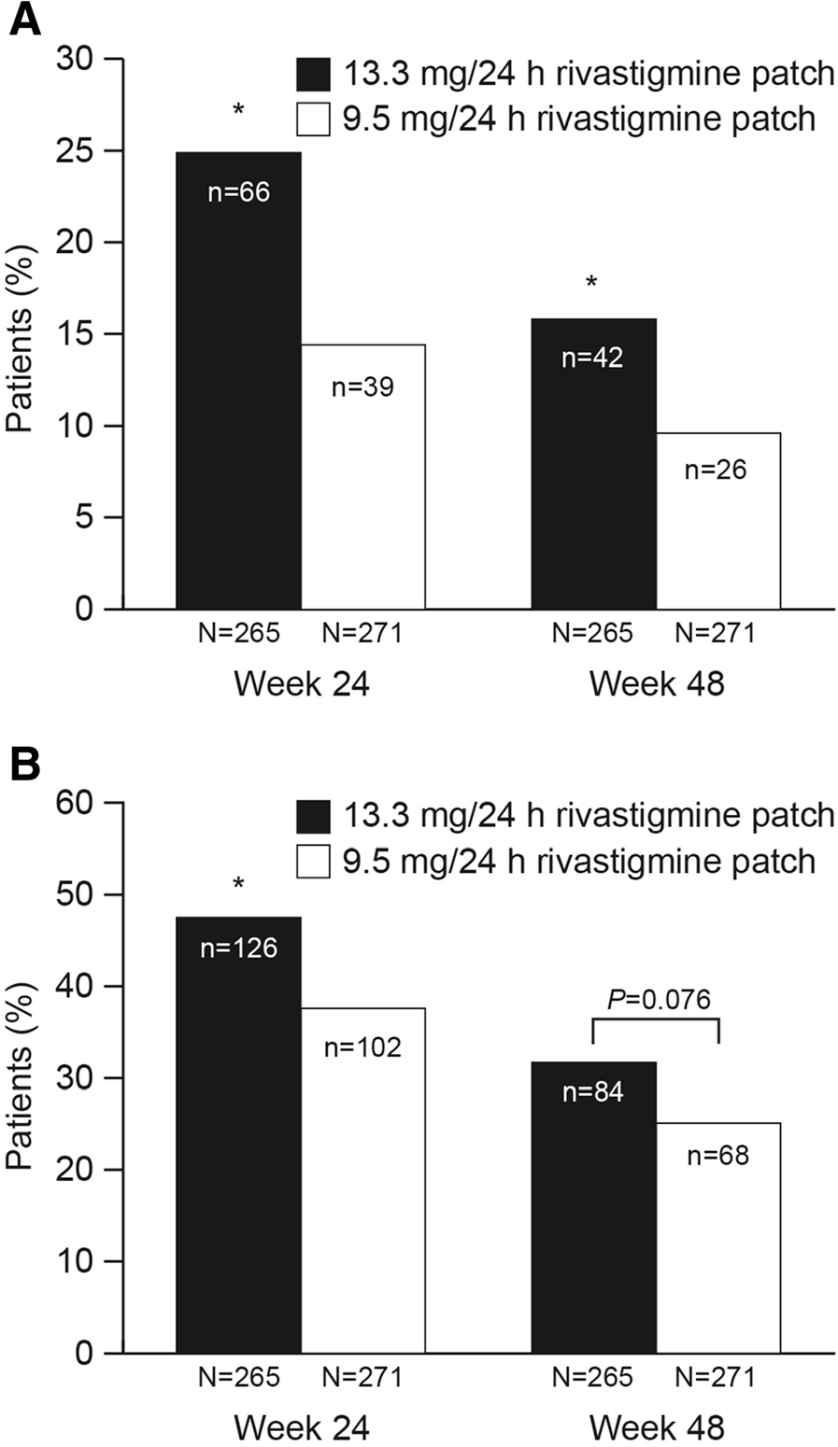
**Responder analyses for the individual assessment scales. (A)** Compared with the 9.5 mg/24 h patch, the 13.3 mg/24 h patch was associated with a significantly higher proportion of patients with ≥4 point improvement on the ADAS-cog at both weeks 24 and 48. *P <0.05, 13.3 mg/24 h versus 9.5 mg/24 h patch. **(B)** Compared with the 9.5 mg/24 h patch, the 13.3 mg/24 h patch was associated with a significantly higher proportion of patients who showed no decline on the ADCS-IADL at Week 24. *P <0.05, 13.3 mg/24 h versus 9.5 mg/24 h patch. ADAS-cog, Alzheimer’s Disease Assessment Scale–cognitive subscale; ADCS-IADL, Instrumental domain of the Alzheimer’s Disease Cooperation Study–Activities of Daily Living scale.

A significantly greater proportion of patients receiving the 13.3 mg/24 h compared with the 9.5 mg/24 h patch demonstrated ≥4 points improvement on the ADAS-cog at Week 24 (n = 66 (24.9%) versus n = 39 (14.4%), *P* = 0.001) and at Week 48 (n = 42 (15.8%) versus n = 26 (9.6%), *P* = 0.020) (Figure 1A). A significantly higher proportion of patients receiving the 13.3 mg/24 h patch compared with the 9.5 mg/24 h patch showed no decline on the ADCS-IADL at Week 24 (n = 126 (47.5%) versus n = 102 (37.6%), *P* = 0.015). The difference at Week 48 was not statistically significant (n = 84 (31.7%) versus n = 68 (25.1%), *P* = 0.076); the difference was numerically in favor of the high-dose 13.3 mg/24 h patch group (Figure 1B).

### Combined response on both the ADAS-cog and ADCS-IADL

Baseline mean scores were comparable between the 13.3 mg/24 h patch and the 9.5 mg/24 h patch groups for the ADAS-cog (34.4 versus 34.9, respectively) and ADCS-IADL (27.5 versus 25.8, respectively). A significantly higher percentage of patients receiving the 13.3 mg/24 h patch compared with those receiving the 9.5 mg/24 h patch were ‘improvers’, demonstrating improvement of ≥4 points on the ADAS-cog plus no decline on the ADCS-IADL, at both Week 24 (n = 44 (16.6%) versus n = 19 (7.0%), *P* <0.001) and Week 48 (n = 21 (7.9%) versus n = 10 (3.7%), *P* = 0.023).

A significantly higher percentage of patients receiving the 13.3 mg/24 h patch compared with those receiving the 9.5 mg/24 h patch were ‘non-decliners’ based on the combined criteria at Week 24 (n = 71 (26.8%) versus n = 44 (16.2%), *P* = 0.002). A trend toward a higher proportion of ‘non-decliners’ was observed with the higher-dose patch compared with the 9.5 mg/24 h patch at Week 48 (n = 36 (13.6%) versus n = 24 (8.9%), *P* = 0.066).

### Predictors of response

All covariates were available for all but four patients, leaving a total of 532 subjects (264 in the 13.3 mg/24 h patch group and 268 in the 9.5 mg/24 h patch group) to be included in the stepwise regression model. Predictors identified as being relevant for a response on the combined ‘improver’ and ‘non-decliner’ criteria are summarized in Table 1.

**Table 1:** **Predictors of response from a stepwise logistic-regression model on combined ‘improver’ criteria and combined ‘non-decliner’ criteria**

**Effect**	**Maximum likelihood estimates**				
	**Parameter**	**Week 24**		**Week 48**	
		**Estimate** ^**a**^	**Pr > ChiSq**	**Estimate** ^**a**^	**Pr > ChiSq**
**‘Improvers’**					
Treatment^b^	Treatment 9.5 mg/24 h patch	−0.580	0.0001	−0.461	0.021
Gender	Female	0.234	0.137	−0.040	0.014
ADAS-cog^c^	ADAS-cog^c^	0.044	0.017	−0.438	0.059
ADCS-IADL^c^	ADCS-IADL^c^	−0.026	0.050	na	na
MMSE^c^	MMSE ≤12^c^	−0.854	<0.0001	na	na
**‘Non-decliners’**					
Treatment^b^	Treatment 9.5 mg/24 h patch	−0.337	0.002	−0.270	0.058
Gender	Female	0.218	0.094	na	na
ADAS-cog^c^	ADAS-cog^c^	0.031	0.014	na	na
ADCS-IADL^c^	ADCS-IADL^c^	na	na	−0.027	0.020
MMSE^c^	MMSE ≤12^c^	−0.497	0.002	−0.351	0.040
Weight, versus >80 kg	Weight 50 to 80 kg	−0.363	0.068^d^	−0.405	0.119^d^
	Weight <50 kg	0.208		0.344	

^a^Positive estimate indicates increased odds of response when increasing the value of the covariate, or when in the given category. Negative estimated effect means reduced odds of response. ^b^Treatment effect compares 9.5 mg/24 h to 13.3 mg/24 h rivastigmine patch. ^c^At DB-BL. ^d^All categories combined. Candidate covariates were: treatment, gender, ADAS-cog score at DB-BL, ADCS-IADL score at DB-BL, MMSE score of ≤12 at DB-BL and weight category (<50, 50 to 80 and >80 kg). ‘Improver’ = patient with an improvement from DB-BL in ADAS-cog score ≥4 points and a change from DB-BL in ADCS-IADL score ≥0 points; ‘non-decliner’ = patient with a change from DB-BL in ADAS-cog score ≤0 points and a change from DB-BL in ADCS-IADL score ≥0 points. ADAS-cog, Alzheimer’s Disease Assessment Scale–cognitive subscale; ADCS-IADL, Instrumental domain of the Alzheimer’s Disease Cooperation Study–Activities of Daily Living scale; DB-BL, double-blind baseline; MMSE, Mini-Mental State Examination; na, not applicable because effect not selected as relevant by the stepwise regression model – only variables (effects) with a *P*-value <0.15 were included in the final model. Treatment was always retained within the model; Pr > ChiSq, *P*-value of the Chi-Square test.

The probability of seeing improvement at Week 24 was estimated to be higher for patients treated with the 13.3 mg/24 h versus the 9.5 mg/24 h patch (*P* = 0.0001). Higher ADAS-cog score and lower ADCS-IADL score at baseline (both indicating more severe impairment) also resulted in an increased estimate of probability of improvement (*P* = 0.017 and 0.050, respectively); conversely, an MMSE score ≤12 at baseline led to a decreased estimate (*P* <0.0001). At Week 48, only male gender and treatment with the 13.3 mg/24 h patch were significant predictors (*P* = 0.014 and 0.021, respectively).

Similarly, the probability of seeing no decline at Week 24 was estimated to be higher for patients treated with the 13.3 mg/24 h versus the 9.5 mg/24 h patch (*P* = 0.002). At Week 48, a baseline ADCS-IADL score indicating a more severe disease status led to an increased estimate of the probability of no decline (*P* = 0.020), while an MMSE score ≤12 at baseline reduced the estimate (*P* = 0.040); treatment with the 13.3 mg/24 h versus the 9.5 mg/24 h patch was borderline non-significant (*P* = 0.058).

## Discussion

According to the present analysis, the proportion of patients demonstrating a clinically relevant response (improvement of at least four points on the ADAS-cog and no decline on the ADCS-IADL) was more than two-fold with the high-dose rivastigmine patch (13.3 mg/24 h) relative to the lower dose (9.5 mg/24 h) at both Week 24 and Week 48. There was a significant difference between dose groups in the proportion of ‘non-decliners’ using the combined analysis (ADAS-cog and ADCS-IADL scales) to Week 24, but this difference was not significant by Week 48. At both time points (Weeks 24 and 48), the proportion of patients classified as responders (showing improvement *or* non-decline) was more than 50% higher with the 13.3 mg/24 h compared with the 9.5 mg/24 h rivastigmine patch.

There was a robust statistical association of the 13.3 mg/24 h rivastigmine patch with a significantly greater likelihood of achieving improvement on combined criteria compared with the 9.5 mg/24 h patch, supporting the clinically meaningful benefit of this higher-dose patch. In a population where all patients steadily decline in cognition and function over the long term, the temporary stability achieved with either dose of rivastigmine patch represents a valuable benefit to patients and their relatives and/or caregivers.

The current analysis provides insights into the primary analysis of the OPTIMA study. The primary study reported deterioration from DB-BL to Week 48 on the ADAS-cog and ADCS-IADL scale with both the 13.3 mg/24 h and the 9.5 mg/24 h patch [12]. Furthermore, in the primary analysis, the 13.3 mg/24 h patch group had a significant benefit (statistically less deterioration) over the 9.5 mg/24 h patch group from Week 16 up to Week 48 on the ADCS-IADL scale, and showed a significant benefit at Week 24 (but not Week 48) using the ADAS-cog. When only ‘improvers’ are considered, this *post-hoc* analysis shows a significant benefit for the 13.3 mg/24 h patch over the 9.5 mg/24 h patch on the ADAS-cog at Week 48. This benefit of the 13.3 mg/24 h patch for the subset of ‘improvers’ was potentially masked by the much larger group of patients who were ‘non-decliners’ or non-responders (that is, showed deterioration) in the primary analysis. The opposite is seen when the effect of treatment on the ADCS-IADL scale is considered. The *post-hoc* analysis (‘non-decliners’) found a statistically significant difference between treatment groups at Week 24, but not at Week 48, as was found in the primary analysis (total population). Therefore, the results from this *post-hoc* analysis offer a valuable opportunity to evaluate the effect of different doses of rivastigmine on different patient types.

To maximize the therapeutic benefit with ChEIs, the optimal dose is tailored to each individual AD patient according to disease stage and other clinical characteristics [1]. Identifying the proportion of patients considered to respond to different doses of ChEI therapy during the course of disease progression, as well as the characteristics of these ‘responders’, will help physicians evaluate the clinical benefit of different treatment regimens and assist them in the choice of optimal therapy for their patients. In this analysis, the proportion of patients who were ‘improvers’ or ‘non-decliners’ was higher at Week 24 than at Week 48 post-randomization on both measurement scales – reflecting the progressive nature of the disease. The mean MMSE score was 14.1 for patients in the 13.3 mg/24 h patch group and 14.2 for patients in the 9.5 mg/24 h patch group, indicating that a large proportion of patients had reached a moderate stage of AD by DB-BL [12].

Predictors of response analyses produced inconsistent results (Table 1). Worse cognition or functional ability at the start of the trial measured by the ADAS-cog or ADCS-IADL, respectively, was associated with a significantly greater chance of improvement (or lack of decline) with rivastigmine after 24 weeks; when patients declined to an MMSE score of less than 12 there was a significantly reduced chance of improvement. Conversely, the probability of response (or absence of decline) after 48 weeks tended to be higher with better cognition at DB-BL (as indicated by either ADAS-cog for improvement, and MMSE category for no-decline, respectively), again accompanied by the opposite effect on the ADCS-IADL. This analysis was limited and biased by the design of the study. Prospectively planned analyses for predictors of response would be a useful approach in clinical trials of agents for treatment of AD.

Reduced function compromises the patient’s ability to live independently, is an important predictor of caregiver burden [15] and is a strong risk factor for nursing home placement or institutionalization [16-18]. In turn, nursing home placement has a marked influence on the overall costs of dementia management [18]. By measuring both cognitive and functional decline, physicians gain a more complete picture of the likely impact of the disease and the available treatments on the patient, their caregiver and the healthcare system in general.

## Conclusions

The therapeutic benefit of the higher-dose 13.3 mg/24 h versus the 9.5 mg/24 h rivastigmine patch derives from a significant increase in the number of ‘improvers’ maintained over 48 weeks. The proportion of patients classified as ‘non-decliners’ (who experienced improvement or no decline) at 48 weeks with the 13.3 mg/24 h patch was also numerically higher than that observed with the 9.5 mg/24 h patch. This stability is an important benefit in a declining population. Predictors of response analyses produced contradictory results that were not easy to interpret. Therefore, further research is warranted.

## Additional file

Additional file 1:
**List of Independent Ethics Committees or Institutional Review Boards.**

## Abbreviations

AD: Alzheimer’s disease
ADAS-cog: Alzheimer’s Disease Assessment Scale–cognitive subscale
ADCS: Alzheimer’s Disease Cooperative Study
ADCS-IADL: Instrumental domain of the ADCS-ADL scale
ADL: activities of daily living
ADRDA: Alzheimer’s Disease and Related Disorders Association
ChEIs: cholinesterase inhibitors
DB: double blind
DB-BL: DB baseline
IOL: initial open label
ITT: intention-to-treat
LOCF: last-observation-carried-forward
MMSE: Mini-Mental State Examination
NINCDS: National Institute of Neurological and Communicative Disorders and Stroke
OPTIMA: OPtimizing Transdermal Exelon In Mild-to-moderate Alzheimer’s disease
OR: odds ratio
SD: standard deviation
