# Supplementary material for: Responder analysis of a randomized comparison of the 13.3 mg/24 h and 9.5 mg/24 h rivastigmine patch
Source: Alzheimers Res Ther. 2015 Mar 8;7(1):9. doi: 10.1186/s13195-014-0088-8 (PMC4353453; doi:10.1186/s13195-014-0088-8)
Supplement: Additional file 1: — List of Independent Ethics Committees or Institutional Review Boards. [file 13195_2014_88_MOESM1_ESM.pdf]

Appendix 16.1.3 List of Independent Ethics Committees or Institutional Review Boards

Center No.	Ethics Committee or Institutional Review Board	Department / Organization	Address Country
101	Sunnybrook Health Sciences Center, IRB		Toronto Ontario M4N 3M5 Canada
103	Centre hospitalier affilie universitaire de Quebec, Reserach Ethics Review Baord		Quebec QC G1J1Z4 Canada
104	Ontario Institutional Review Board		Aurora Ontario L4G 0A5 Canada
105	Centre de santé Elisabeth-Bruyere Health Centre		Ottawa Ontario K1N 5C8 Canada
107	McGill University IRB		Montreal Quebec H3G 1Y7 Canada
108	Institutional Review Board Services		Aurora Ontario L4G 0A5 Canada
109	University of Alberta, Health Research Ethics Board		Edmonton Alberta T6G 2S2 Canada
110	Capital Health Research Ethics Board	Room 118, Centre for Clinical Research	Halifax NS B3H 1V7 Canada
111	Institutional Review Board Services		Aurora Ontario L4G 0A5 Canada
112	Institutional Review Board Services		Aurora Ontario L4G 0A5 Canada
114	The University of British Columbia, Office of Research Services, Clinical Research Ethics Board		Vancouver BC V5Z 1L8 Canada
115	Comité d'èthique de la recherche de I'MMR	Pavillon Rachel Tourigny	Montréal Québec H1T 2M4 Canada
116	University of Saskatchewan, Research Ethics Office		Saskatoon SK S7N 4J8 Canada
117	Institutional Reveiw Board Services		Aurora Ontario L4G 0A5 Canada
202	CPP Est-III Hopital de Brabois Batiment de l''institu de Formation en Soins Infirmiers		Vandoeuvre Les Nancy Cedex 54511 France
203	CPP Est-III Hopital de Brabois Batiment de l''institu de Formation en Soins Infirmiers		Vandoeuvre Les Nancy Cedex 54511 France
204	CPP Est-III Hopital de Brabois Batiment de l''institu de Formation en Soins Infirmiers		Vandoeuvre Les Nancy Cedex 54511 France

This document (090095af8305e493 in docbase CREDI_EH) has been digitally signed with external signatures using Entrust PKI.
Signatures manifested as of 10/11/2011 11:50:24 AM, signing status at this time: Completed (1 of 1 signatures)
Approved for report publication by Downs Pamela in East Hanover at Tue, 11 Oct 2011 07:49:15 AM EDT

Center No.	Ethics Committee or Institutional Review Board	Department / Organization	Address Country
205	CPP Est-III<br>Hopital de Brabois<br>Batiment de l'"institu de<br>Formation en Soins Infirmiers		Vandoeuvre Les Nancy Cedex<br>54511<br>France
206	CPP Est-III<br>Hopital de Brabois<br>Batiment de l'"institu de<br>Formation en Soins Infirmiers		Vandoeuvre Les Nancy Cedex<br>54511<br>France
207	CPP Est-III<br>Hopital de Brabois<br>Batiment de l'"institu de<br>Formation en Soins Infirmiers		Vandoeuvre Les Nancy Cedex<br>54511<br>France
208	CPP Est-III<br>Hopital de Brabois<br>Batiment de l'"institu de<br>Formation en Soins Infirmiers		Vandoeuvre Les Nancy Cedex<br>54511<br>France
209	CPP Est-III<br>Hopital de Brabois<br>Batiment de l'"institu de<br>Formation en Soins Infirmiers		Vandoeuvre Les Nancy Cedex<br>54511<br>France
210	CPP Est-III<br>Hopital de Brabois<br>Batiment de l'"institu de<br>Formation en Soins Infirmiers		Vandoeuvre Les Nancy Cedex<br>54511<br>France
211	CPP Est-III<br>Hopital de Brabois<br>Batiment de l'"institu de<br>Formation en Soins Infirmiers		Vandoeuvre Les Nancy Cedex<br>54511<br>France
212	CPP Est-III<br>Hopital de Brabois<br>Batiment de l'"institu de<br>Formation en Soins Infirmiers		Vandoeuvre Les Nancy Cedex<br>54511<br>France
213	CPP Est-III<br>Hopital de Brabois<br>Batiment de l'"institu de<br>Formation en Soins Infirmiers		Vandoeuvre Les Nancy Cedex<br>54511<br>France
214	CPP Est-III<br>Hopital de Brabois<br>Batiment de l'"institu de<br>Formation en Soins Infirmiers		Vandoeuvre Les Nancy Cedex<br>54511<br>France
301	Federfuhrende Ethik-Kommission	Med. Ethik-Kommission II	Mannheim 68169<br>Germany
302	Ernst-Moritz-Arndt-Universität	Ethik-Kommission	Greifswald 17487<br>Germany
303	Otto-von-Guericke-Universität Magdeburg	Ethik-Kommission	Magdeburg 39120<br>Germany
305	Landesamt fur Gesundheit und Soziales	Geschäftsstelle der Ethik-Kommission	Berlin 10707<br>Germany
306	Landesamt fur Gesundheit und Soziales	Geschäftsstelle der Ethik-Kommission	Berlin 10707<br>Germany

This document (090095af8305e493 in docbase CREDI_EH) has been digitally signed with external signatures using Entrust PKI.
Signatures manifested as of 10/11/2011 11:50:24 AM, signing status at this time: Completed (1 of 1 signatures)
Approved for report publication by Downs Pamela in East Hanover at Tue, 11 Oct 2011 07:49:15 AM EDT

Center No.	Ethics Committee or Institutional Review Board	Department / Organization	Address Country
307	Ethik-Kommission	an der Mediznischen Fakultät Universität Leipzig	Leipzig 04107 Germany
309	Ärztekammer Nordrhein	Ethik-Kommission	Dűsseldorf 40474 Germany
310	Ethikommission der Medizinischen Fakultat der Heinrich	Heine-Universitat Dusseldorf	Dusseldorf 40225 Germany
311	Ärztekammer Nordrhein	Ethik-Kommission	Dusseldorf 40474 Germany
312	Geschäftsselle Ethikommission	Universität zu Koln	Koln 50937 Germany
313	Rheinische Friedrich-Wilhelms-Universität	Ethik-Kommission	Bonn 53105 Germany
316	Ethik-Kommission	Johann Wolfgang Goethe-Universitat	Frankfurt am Main 60590 Germany
317	Bayerische Landesärztekammer	Ethik-Kommission	Munchen 81677 Germany
318	Medizinische Ethik-Kommission II der Fakulät für Kinische Medizin Mannheim		Mannheim 68169 Germany
319	Bayerische Landesärztekammer	Ethik-Kommission	Munchen 81677 Germany
320	Beteiligte Ethik-Kommissionen	Ethik-Kommission	Stuttgart 70597 Germany
321	Bayerische Landesärztekammer	Ethik-Kommission	Munchen 81677 Germany
322	Medizinische Ethik-Kommission II der Fakulät für Kinische Medizin Mannheim		Mannheim 68169 Germany
323	Bayerische Landesärztekammer	Ethik-Kommission	Munchen 81677 Germany
325	Medizinische Ethik-Kommission II der Fakulät für Kinische Medizin Mannheim		Mannheim 68169 Germany
326	Ärztekammer Niedersachsen	Ethik-Kommission	Hannover 30175 Germany
327	Landesärztekammer Hessen	Ethik-Kommission	Frankfurt am main 60488 Germany
328	Landesärztekammer Brandenburg	Ethik-Kommission	Cottbus 03044 Germany
329	Ruhr-Universität Bochum	Ethik-Kommission	Bochum 44789 Germany
401	Comitato Etico Dell'ircca Ospedale	Maggiore Policlinco, Mangiagalli e Regina Elena di Milano	Milano 20122 Italy
402	Comitato Etico Interaziendale Delle Aso S. Giovanni Battista E CTO/CRF/Maria Adelaide Di Torino		Torino 10126 Italy

This document (090095af8305e493 in docbase CREDI_EH) has been digitally signed with external signatures using Entrust PKI.
Signatures manifested as of 10/11/2011 11:50:24 AM, signing status at this time: Completed (1 of 1 signatures)
Approved for report publication by Downs Pamela in East Hanover at Tue, 11 Oct 2011 07:49:15 AM EDT

Center No.	Ethics Committee or Institutional Review Board	Department / Organization	Address Country
404	Comitato Etico Per Speriment Azione Dei Medicinali Della		Arezzo 52100 Italy
405	Comitato Etico Istituzoni	Ospedaliere Cattoliche (Ceioc) di Brescia	Brescia 25125 Italy
406	Comitato Etico Per La Sperimentazione Clinical Dei Medicinali		Firenze 50139 Italy
407	Comitato Etico Indipendente Della Asl Roma C-Lazio		Roma 00144 Italy
408	Comitato Etico Dell'Azienda Ospedaliera G. Salvini Di Garbagnate Milanese-Garbagnate		Milanese 20024 Italy
409	Comitato Etico Per La Sperimentazione Dell'Azienda		Verona 37134 Italy
410	Comitato Etico Delle Aziende Sanitarie Dell'Umbria-Segreteria		Corciano 06073 Italy
411	Comitato Etico Della Asl To/2 Di Torino		Torino 10154 Italy
412	Comitato Etico Della Provincia Di Ferrara		Ferrara 44100 Italy
413	Comitato Etico Dell'Ausl Di Bologna		Bologna 40124 Italy
414	Comitato Etico Dell'Ente Ospedaliero Ospedali Galliera Di Genova		Genova 16128 Italy
415	Comitato Etico Dell'Ospedale Sant' Andrea Di Roma		Roma 00189 Italy
416	Comitato Etico Dell' Univerista' Cattolica del S. Cuore		Roma 00198 Italy
418	Comitato Etico Dell'Azienda Policlinico Umberto I Di Roma		Roma 00161 Italy
419	Comitato Etico Dell'Irccs Fondazione Istituto Neurologico Casmiro Mondino Di Pavia		Pavia 27100 Italy
420	Comitato Etico Dell'IRCCS Fondazione San Raffaele Del Monte Tabor Di Milano		Milano 20132 Italy
421	Comiitatp Ethico Dell'Asl 5 Spezzine di La Spezia		La Spezia 191214 Italy
423	Comitato Di Bioetica Dell'Inrca Istituto Nazionale Di Riposo e Cura Per Anziani Di Ancona		Ancona 60131 Italy

This document (090095af8305e493 in docbase CREDI_EH) has been digitally signed with external signatures using Entrust PKI.
Signatures manifested as of 10/11/2011 11:50:24 AM, signing status at this time: Completed (1 of 1 signatures)
Approved for report publication by Downs Pamela in East Hanover at Tue, 11 Oct 2011 07:49:15 AM EDT

Center No.	Ethics Committee or Institutional Review Board	Department / Organization	Address Country
424	Comitato Etico Delle Aziende Sanitarie Dell'Umbria Segreteria Scientifico Amministrativa Del Ceas Umbria		Corciano 06073 Italy
425	Regione Veneto Azienda Ospedaliera Di Padova, Comitato Etico per la Sperimentazione		Padova 35128 Italy
426	Comitato Di Bioetica Dell'Inrca Istituto Nazionale Di Riposo e Cura Per Anziani Di Ancona Via Della Montagnola 164		Ancona 60131 Italy
427	Comitato Etico Dell'Azienda Ospedaliero-Universitaria Pisana		Pisa 56126 Italy
428	Comitato Etico Dell'Ausl 8 Di Calgiari		Cagliari 09127 Italy
429	Comitato Etico Della Provincia di Modena		Modena 41100 Italy
430	Comitato Etico dell'Azienda Ospedaliera Policinico Consorziale		Bari 70124 Italy
431	Comitato Etico dell'Azienda Ospedaliera Universitaria Polclinico tor Vergata		Roma 00133 Italy
432	Comitato Etico c/o Framacia		Napoli 80100 Italy
433	Comitato Etico Azienda Ospedaliera Garibaldi-San Luigi- Curro-Ascoli- Tomaselli		Catania 95125 Italy
434	Comitato di Bioetica dell'Inrca Istituto Nazionale Di Riposo e Cura per Anziani di Ancona		Ancona 60131 Italy
435	Comitato Etico dell'Azieda Ospedaliero - Universitaria Ospedali Riuniti di Foggia		Foggia 71100 Italy
436	Comitato Etico ASL di Brescia		Brescia 25128 Italy
437	Comitato Etico della ASL della Provincia de Cremona		Cremona 26100 Italy
501	Quorum Review Inc.		Seattle WA 98101 USA
502	Quorum Review Inc.		Seattle WA 98101 USA
503	Quorum Review, Inc.		Seattle WA 98101 USA
504	Hunterdon Medical Center Institutional Review Board		Flemington NJ 08822 USA

This document (090095af8305e493 in docbase CREDI_EH) has been digitally signed with external signatures using Entrust PKI.
Signatures manifested as of 10/11/2011 11:50:24 AM, signing status at this time: Completed (1 of 1 signatures)
Approved for report publication by Downs Pamela in East Hanover at Tue, 11 Oct 2011 07:49:15 AM EDT

Center No.	Ethics Committee or Institutional Review Board	Department / Organization	Address Country
505	Quorum Review Inc.		Seattle WA 98101<br>USA
506	Quorum Review Inc.		Seattle WA 98201<br>USA
507	Quorum Review Inc.		Seattle WA 98101<br>USA
508	Quorum Review Inc.		Seattle WA 98101<br>USA
509	Mercer University		Macon GA 31207<br>USA
510	Quorum Review Inc.		Seattle WA 98101<br>USA
511	IRB-Mount Sinai Medical Center		Miami Beach FL 33140<br>USA
512	Quorum Review Inc.		Seattle WA 98101<br>USA
513	Florida Atlantic University IRB		Boca Raton FL 33431<br>USA
515	Quorum Review, Inc.		Seattle WA 98101<br>USA
517	Quorum Review, Inc.		Seattle WA 98101<br>USA
518	Western Institutional Review Board		Olympia WA 98502-5010<br>USA
519	Quorum Review, Inc.		Seattle WA 98101<br>USA
520	Quorum Review, Inc.		Seattle WA 98101<br>USA
521	Quorum Review, Inc.		Seattle WA 98101<br>USA
522	Quorum Review, Inc.		Seattle WA 98101<br>USA
523	Quorum Review, Inc.		Seattle WA 98101<br>USA
524	Quorum Review, Inc.		Seattle WA 98101<br>USA
525	Quorum Review, Inc.		Seattle WA 98101<br>USA
526	Quorum Review, Inc.		Seattle WA 98101<br>USA
527	Quorum Review, Inc.		Seattle WA 98101<br>USA
528	Quorum Review, Inc.		Seattle WA 98101<br>USA
529	California Pacific Medical Center Research Institute-IRB		San Francisco CA 94120<br>USA
530	Quorum Review, Inc.		Seattle WA 98101<br>USA

This document (090095af8305e493 in docbase CREDI_EH) has been digitally signed with external signatures using Entrust PKI.
Signatures manifested as of 10/11/2011 11:50:24 AM, signing status at this time: Completed (1 of 1 signatures)
Approved for report publication by Downs Pamela in East Hanover at Tue, 11 Oct 2011 07:49:15 AM EDT

Center No.	Ethics Committee or Institutional Review Board	Department / Organization	Address Country
531	Quorum Review, Inc.		Seattle WA 98101<br>USA
532	Quorum Review, Inc.		Seattle WA 98101<br>USA
533	Washington University School of Medicine<br>Human Reserach Protection Office		St. Louis MO 63110<br>USA
534	University of North Texas Health Science Center		Fort Worth TX<br>USA
535	Sun Health Institutional Review Board		Sun City AZ 85351<br>USA
536	Quorum Review, Inc.		Seattle WA 98101<br>USA
537	St. John's Mercy Medical Center IRB		St. Louis MO 63141<br>USA
538	Texas Health Resources IRB		Dallas TX 75231<br>USA
539	Quorum Review, Inc.		Seattle WA 98101<br>USA
540	Quorum Review, Inc.		Seattle WA 98101<br>USA
541	Quorum Review, Inc.		Seattle WA 98101<br>USA
543	Quorum Review, Inc.		Seattle WA 98101<br>USA
544	Quorum Review, Inc.		Seattle WA 98101<br>USA
545	Quorum Review, Inc.		Seattle WA 98101<br>USA
546	Quorum Review, Inc.		Seattle WA 98101<br>USA
547	Quorum Review, Inc.		Seattle WA 98101<br>USA
548	Quorum Review, Inc.		Seattle WA 98101<br>USA
549	Quorum Review, Inc.		Seattle WA 98101<br>USA
550	Quorum Review, Inc.		Seattle WA 98101<br>USA
551	Western Institutional Review Board		Olympia WA 98508<br>USA
552	Institutional Review Board of St. John Hospital and Medical Center		Detroit MI 48236<br>USA
553	Office for the protection of Research Subjects		Chicago IL 60611<br>USA
554	Quorum Review, Inc.		Seattle WA 98101<br>USA

This document (090095af8305e493 in docbase CREDI_EH) has been digitally signed with external signatures using Entrust PKI.
Signatures manifested as of 10/11/2011 11:50:24 AM, signing status at this time: Completed (1 of 1 signatures)
Approved for report publication by Downs Pamela in East Hanover at Tue, 11 Oct 2011 07:49:15 AM EDT

Center No.	Ethics Committee or Institutional Review Board	Department / Organization	Address Country
555	Quorum Review, Inc.		Seattle WA 98101<br>USA
556	Quorum Review, Inc.		Seattle WA 98101<br>USA
557	Quorum Review, Inc.		Seattle WA 98101<br>USA
558	Quorum Review, Inc.		Seattle WA 98101<br>USA
559	Quorum Review, Inc.		Seattle WA 98101<br>USA
560	Quorum Review, Inc.		Seattle WA 98101<br>USA
561	Providence Portland Medical Center		Portland OR 97213<br>USA
562	Marshall University IRB		Huntington WV 25701<br>USA
563	Quorum Review, Inc.		Seattle WA 98101<br>USA
564	Quorum Review, Inc.		Seattle WA 98101<br>USA
566	Robert C. Byrd Health Sciences Center		Charleston WV 25304<br>USA
567	Quorum Review, Inc.		Seattle WA 98101<br>USA
568	Quorum Review, Inc.		Seattle WA 98101<br>USA
570	Quorum Review, Inc.		Seattle WA 98101<br>USA
601	Comite Etico De Investigacion Clinica<br>C/ Doctor Aiguder, n° 88 1ª Planta<br>Institut Municipal D'Investigacio Medica edificio PRBB		Barcelona 08003<br>Spain
602	Agencia de Ensayo Clinicos - Servicio de Farmacia<br>Comite de Etico de Investigacion Clinica<br>Hospital Clinic i Provincial<br>C/Villarroel, 170 Sotano, escalera 8		Barcelona 08036<br>Spain
603	Comite Etico de Investigacion Clinica Illes Baleares		Palma de Mallorca 07003<br>Spain
621	Ethikkommission beider Basel / EKBB		Basel 4056<br>Switzerland
622	Comitato etico cantonale c/o Sezione sanitaria		Bellinzona CH-6500<br>Switzerland
623	Ethikkommission beider Basel / EKBB		Basel 4031<br>Switzerland

This document (090095af8305e493 in docbase CREDI_EH) has been digitally signed with external signatures using Entrust PKI.
Signatures manifested as of 10/11/2011 11:50:24 AM, signing status at this time: Completed (1 of 1 signatures)
Approved for report publication by Downs Pamela in East Hanover at Tue, 11 Oct 2011 07:49:15 AM EDT
